# Asymmetry of attentive networks contributes to adult Attention-deficit/hyperactivity disorder (ADHD) pathophysiology

**DOI:** 10.1007/s00406-024-01927-4

**Published:** 2024-11-02

**Authors:** Valeria Parlatini, Joaquim Radua, Naianna Robertsson, Alessandra Lintas, Emel Atuk, Flavio dell’Acqua, Michel Thiebaut de Schotten, Declan Murphy

**Affiliations:** 1 Institute of Translational Neurodevelopment, Department of Forensic and Neurodevelopmental Sciences, Institute of Psychiatry, Psychology and Neuroscience, King’s College London, London, SE5 8AF UK; 2 Department of Forensic and Neurodevelopmental Sciences, Institute of Psychiatry, Psychology and Neuroscience, King’s College London, 16 De Crespigny Park, London, SE5 8AF UK; 3 Centre for Innovation in Mental Health, School of Psychology, University of Southampton, Southampton, UK; 4 Solent NHS Trust, Southampton, UK; 5 Imaging of Mood and Anxiety-Related Disorders (IMARD) Group, Institut d’Investigacions Biomèdiques August Pi I Sunyer (IDIBAPS), Campus Casanova, Casanova, 143, 08036 Barcelona, Spain; 6 Neuroheuristic Research Group, HEC Lausanne, University of Lausanne, UNIL-Chamberonne, 1015 Lausanne, Quartier Switzerland; 7 Sussex Partnership NHS Foundation Trust, Dartford, DA1 2EN UK; 8 Department of Neuroimaging, Institute of Psychiatry, Psychology and Neuroscience, King’s College London, London, SE5 8AF UK; 9 Institute of Psychiatry, NIHR Biomedical Research Centre for Mental Health at South London and Maudsley NHS Foundation Trust and King’s College London, London, SE5 8AF UK; 10 Brain Connectivity and Behaviour Group, Sorbonne Universities, Paris, France; 11 Groupe d’Imagerie Neurofonctionnelle, Institut des Maladies Neurodégénératives-UMR 5293, CNRS, CEA University of Bordeaux, Bordeaux, France

**Keywords:** Attention-deficit/hyperactivity disorder (ADHD), Diffusion weighted imaging (DWI), Tractography, Superior longitudinal fasciculus (SLF), Methylphenidate

## Abstract

Diffusion imaging studies in Attention-deficit/hyperactivity disorder (ADHD) have revealed alterations in anatomical brain connections, such as the fronto-parietal connection known as superior longitudinal fasciculus (SLF). Studies in neurotypical adults have shown that the three SLF branches (SLF I, II, III) support distinct brain functions, such as attention and inhibition; and that their pattern of lateralization is associated with attention performance. However, most studies in ADHD have investigated the SLF as a single bundle and in children; thus, the potential contribution of the lateralization of the SLF branches to adult ADHD pathophysiology remains to be elucidated. We used diffusion-weighted spherical deconvolution tractography to dissect the SLF branches in 60 adults with ADHD (including 26 responders and 34 non-responders to methylphenidate, MPH) and 20 controls. Volume and hindrance modulated orientational anisotropy (HMOA), which respectively reflect white matter macro- and microstructure, were extracted to calculate the corresponding lateralization indices. We tested whether neurotypical controls differed from adults with ADHD, and from treatment response groups in sensitivity analyses; and investigated associations with clinico-neuropsychological profiles. All the three SLF branches were lateralized in adults with ADHD, but not in controls. The lateralization of the SLF I HMOA was associated with performance at the line bisection, not that of the SLF II volume as previously reported in controls. Further, an increased left-lateralization of the SLF I HMOA was associated with higher hyperactivity levels in the ADHD group. Thus, an altered asymmetry of the SLF, perhaps especially of the dorsal branch, may contribute to adult ADHD pathophysiology.

**Supplementary Information:**

The online version contains supplementary material available at 10.1007/s00406-024-01927-4.

## Introduction

Attention-deficit/hyperactive disorder (ADHD) is a neurodevelopmental condition characterized by inattentive and/or hyperactive-impulsive symptoms [1], and often accompanied by neuropsychological deficits, especially in executive functions such as attention, working memory, ad response inhibition [2]. It is estimated to affect about 5% of children, mainly males, and to persist in about 40–50% of adults [3]. Higher rates of academic and occupational failure, drug use, and legal offences have been observed in adult ADHD cohorts, and associated with high cost for society [2]. Stimulant medication, such as methylphenidate (MPH), represents the first line treatment and are generally effective in reducing ADHD core symptoms, but adults have lower response rates than children [4, 5]. Therefore, we need to better understand the neurobiological characteristics underlying symptoms, associated neuropsychological deficits, and treatment response in adults.

Prior neuroimaging studies have identified diffuse brain anatomical and functional alterations in individuals with ADHD, especially in fronto-striatal-parietal-cerebellar regions and their connections [6–9]. The anatomy of brain connections can be investigated using diffusion weighted imaging (DWI), which allows the identification of white matter bundles and the measure of their microstructural properties [10, 11]. As highlighted in a recent systematic review of DWI studies in ADHD, most studies published so far were in children and focused on fronto-striatal connections, in line with a dominant pathophysiological hypothesis of this condition [9]. Thirty-two studies in children/adolescents and 10 studies in adult/mixed samples reported fronto-striatal tract metric alterations in participants with ADHD, which were associated with symptom severity but also executive dysfunction and poor schooling. Nevertheless, the meta-analysis of 25 tract-based spatial statistics (TBSS) studies pointed to more consistent alterations in the splenium and body of the corpus callosum, extending to the cingulum. These posterior interhemispheric connections subserve cognitive and motor functions affected in ADHD, including working memory, cognitive performance, and motor control. Of note, the meta-regression analysis revealed that these alterations were related to older age, and case–control differences did not survive in the meta-analysis including only studies in children. The lack of findings in children was suggested to be related to the late development of white matter tracts, which may increase case–control differences in adulthood [9]. This work also highlighted that other relevant brain networks have received less attention, especially in adults. For instance, one of the largest association systems in the human brain is represented by the superior longitudinal fasciculus (SLF), which connects fronto-parietal regions [12]. Most prior studies in ADHD reported reduced SLF fractional anisotropy (FA), a measure of white matter microstructural organization, which was associated with symptom severity and cognitive deficits, such as executive dysfunction and poor memory [9]. However, these prior studies mostly included children and findings may not necessarily apply to the adult ADHD population.

Further, advances in diffusion weighted tractography algorithms are increasingly allowing researchers to disentangle subcomponents of larger white matter tracts [13]. This offers the invaluable opportunity to also disentangle their potential differential contribution to the pathophysiology of ADHD. For instance, there is evidence that the SLF can be subdivided into three branches (SLF I, II, III)[14]; however, most prior studies dissected the SLF as a single bundle and did not investigate the pattern of lateralization of the SLF branches [15–19].

Investigating the potential differential role of the SLF branches and their pattern of lateralization in adult ADHD is of relevance for several reasons. First, although there is not absolute consensus on the anatomy and functional roles of the SLF branches [12], prior work has shown that they are differentially implicated in brain functions affected in ADHD, from attention to motor inhibition [14, 20, 21]. The dorsal SLF (SLF I), which corresponds to the dorsal attentive network (DAN), connects superior parietal and frontal regions and is involved in the processing of spatial/motor information, such as voluntary oriented attention. The ventral SLF (SLF III), which corresponds to the ventral attentive network (VAN), connects inferior parietal and frontal regions and supports functions such as automatically captured attention and response inhibition. Finally, the SLF II supports the communication between the SLF I and III and further contributes to attentive functions, but also serves brain regions with flexible response properties [20]. Second, prior work has shown that the pattern of lateralization of the SLF branches was associated with variation in visuospatial attention in neurotypical adults [14]. Specifically, a greater right-lateralization of the SLF II volume was associated with greater left deviation at the line bisection, a test of attentional bias for one visual hemifield [14]. These findings reflect the right-sided dominance of attentive processes [22, 23]. Finally, there is preliminary evidence that pre-treatment brain connectivity characteristics may be associated with variation in treatment response in ADHD [24, 25], including the pattern of lateralization of the SLF I volume. Specifically, our prior study identified an increased right-lateralization of the SLF I volume in MPH treatment-resistant adults (but not in treatment responders) as compared to neurotypical controls [24]. These preliminary findings indicate that a more comprehensive analysis of potential differences between responders/non-responders and controls is warranted, also including the other two SLF branches and measures of lateralization of microstructural organization.

Taken together, these prior findings suggest that the pattern of lateralization of the SLF branches may potentially contribute to adult ADHD pathophysiology. However, it is unknown whether adults with ADHD differ from neurotypical controls in their pattern of lateralization of the SLF branches, and whether this is associated with their clinical severity and neuropsychological profiles. To address these questions, we investigated the pattern of lateralization of the three SLF branches in 60 adults with ADHD (including 26 responders and 34 non-responders to MPH) and 20 controls using spherical deconvolution tractography. We then compared controls with the whole ADHD sample, and with responders and non-responders to MPH in sensitivity analyses. Finally, we analyzed associations between SLF lateralization and clinico-neuropsychological profiles.

## Methods

### Sample

Power calculation and sample characteristics have been previously described [24]. In brief, we recruited 60 adults from the Adult ADHD Clinic, Maudsley Hospital (London, UK), who met DSM-V diagnostic criteria for ADHD, were aged 18–45, had intelligence quotient (IQ) above 70, and no current clinically diagnosed comorbidity. We only included males to enhance sample homogeneity, because ADHD is more commonly diagnosed in males [26], and there is preliminary evidence of sex-related differences in brain connectivity [27–30] and response to stimulant treatment [31–33]. Please see Discussion for potential limitations. We mainly aimed at recruiting medication-naïve individuals and, although a minority was previously treated with ADHD medication (see Results), none received psychopharmacological treatment for at least a year before the study. Finally, 20 neurotypical controls matched for IQ, age, and sex provided baseline DWI scans for comparative analyses.

### Research protocol

This study is part of a larger trial using multiple imaging modalities and a single-blind placebo-controlled cross-over design, followed by a longitudinal open-label phase (NCT 03709940). The trial investigated whether pre-treatment brain characteristics (under a single dose of MPH or placebo) were associated with the clinical response to two-month MPH-treatment in adult ADHD. The full research protocol is described in [24]. This specific study does not investigate ‘predictors’ of treatment response (reported in [24]), but the pattern of lateralization of the SLF branches and their anatomo-clinical correlations. Sixty male adults with ADHD completed clinical and neuropsychological evaluations under placebo (baseline) and under a single dose of 20 mg immediate-release MPH (acute MPH). The MPH dose was selected as previously shown to affect brain activation during functional magnetic resonance imaging (fMRI) tasks in adults [34]. Evaluations included the Barkley Adult ADHD Rating Scale-IV (BAARS-IV) (Barkley, 2011), the Line bisection [35], and the Quantitative behavior (Qb) test (https://www.qbtech.com). Please see supplementary material (page 2) for a description of each measure and their rationale. ADHD participants also underwent baseline DWI scanning before starting treatment with the same long-acting formulation of MPH (Concerta XL, titrated up to 54 mg/day according to usual clinical care). Treatment response was measured at two months (follow-up) using clinical and behavioral measures, as previously reported [24] (see also supplementary material, page 3). All participants provided written consent. The study was approved by Camden and Islington Research Ethics Committee (REC number 12/LO/0630) and complied with the Helsinki Declaration and ethical standards on human experimentation.

### Diffusion MRI data acquisition, preprocessing and tractography

Diffusion imaging data acquisition parameters, preprocessing and tract dissections have been previously described [24]. For each of the SLF branches, we extracted two metrics, one indicative of the microstructural organization, i.e. the Hindrance Modulated Orientational Anisotropy (HMOA) [36], and one reflecting the macrostructural organization, i.e. the volume. We selected MHOA as it provides similar information as FA in classical diffusion tensor imaging (DTI), but can be more reliably used in brain regions with complex fiber organization, such as those crossed by the SLF, and thus may be more sensitive to changes in white matter properties, such as fiber size, axonal number, and myelination [36]. Volume refers to the total volume of voxels intersected by the streamlines of a single tract, thus reflects the size of a white matter tract and is related to parameters such as the number and size of axons, as well as myelination, which affect conduction speed [37, 38]. We selected this measure because the asymmetry of the SLF II volumes has been previously associated with visuospatial attention performance in neurotypical adults [14]. We used HMOA and volume to calculate the respective Lateralization Indices (LIs) for each pair of the SLF branches, according to the formula: (Right metric–Left metric)/(Right metric + Left metric). Positive values reflect a rightward asymmetry, whereas negative values indicate a leftward asymmetry [14]. Imaging data will be deposited in a publicly available repository upon publication.

### Statistical analysis

We used SPSS software (v26, IBM) to confirm tract metric normality, through histograms and Q-Q plots, and to carry out the statistical analyses.

#### Line bisection

We ran a one-sample t-test to verify whether the mean deviation at the line bisection was significantly different from zero at baseline, and a paired t-test to investigate potential changes in mean deviation under a single dose of MPH (as compared to baseline).

#### Pattern of lateralization of the SLF branches

To assess whether tract volume or HMOA of one or more SLF branches were lateralized, we ran a one-sample omnibus test for each metric. This consists of a linear model with the branch as a within-subject covariate of no interest, separately for ADHD participants and controls. Post-hoc t-tests were computed to identify which branch was significantly lateralized. An omnibus test refers to the F Test of a one-sample repeated-measures ANOVA (note that there are three pairs of branches and thus three lateralization indices per individual). This tests the overall null hypothesis that the SLF branches are not lateralized, whereas the alternative hypothesis is that the lateralization index of at least one SLF branch is significantly different from zero. This approach offers the advantage of performing a single test for the three pairs of branches. If the null hypothesis is rejected, post-hoc t-tests are then run to determine which SLF branch or branches are significantly lateralized.

#### Group comparisons

To compare the overall lateralization in adults with ADHD and controls, we ran a two-way omnibus test for each metric, consisting of a linear model with the branch as a within-subject covariate of no interest and the groups as a between-subject factor. Post-hoc t-tests were computed to identify the branches whose lateralization index was significantly different between ADHD participants and controls. We also ran two sensitivity analyses for each tract metric (volume and HMOA) to compare controls with either responders or non-responders to two-month MPH treatment.

#### Correlations between lateralization and clinico-neuropsychological profiles

Finally, we ran correlation analyses to investigate the association between the lateralization pattern of the SLF branches and clinico-neuropsychological profiles in the whole ADHD sample. We considered symptom severity, mean deviation at the line bisection, and Qb test parameters as described in supplementary material (page 2). Specifically, we considered baseline clinico-neuropsychological measures; their change under a single dose of MPH (as compared to baseline); and their change at follow-up (as compared to baseline). We applied Bonferroni correction for multiple comparisons (three pairs of SLF branches, p ≤ 0.017).

#### Secondary analyses

To further explore the potential relationship between SLF lateralization and individual characteristics related to neuropsychological performance, we ran correlations between the lateralization indices and total IQ and handedness. Please see Supplementary material (page 2) for details on measures. Finally, to better understand whether the corresponding lateralization indices based on volume and HMOA reflected partly different white matter properties, we ran correlations among indices, and calculated a composite lateralization score, defined as (SLF LI volume + SLF LI HMOA)/2, for each of the three branches and repeated the group comparisons and correlations. We applied Bonferroni correction for multiple comparisons (N of comparisons = 3, p ≤ 0.017).

## Results

### Sample

The sample included 60 male adults with ADHD, among whom 26 were classified as responders and 34 as non-responders to MPH. The two groups did not significantly differ in age, handedness, total IQ, ADHD presentation, and MPH dose at follow-up. However, non-responders had significantly lower levels of baseline total ADHD symptom severity as measured by the BAARS-IV. Full statistical results are reported in [24].

### Statistical analysis

#### Line bisection

At a group level, ADHD subjects deviated toward the left in the line bisection at baseline (mean = -0.159 SD = 0.247; t(59) = − 5.013, p < 0.001). This left deviation reduced under a single dose of MPH but the difference was not significant (mean = -0.125, SD = 0.274); t(59) = − 1.344, p = 0.184).

#### Pattern of lateralization of the SLF branches

**Fig. 1:**
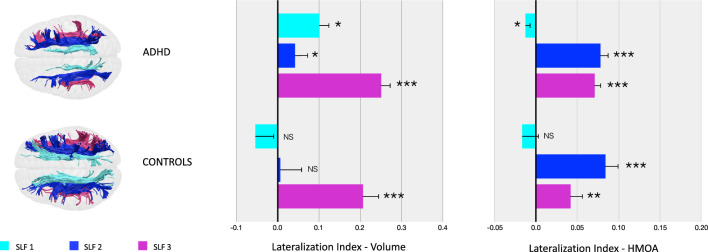
Lateralization of the SLF branches in ADHD participants and controls. Means and confidence intervals for each SLF branch lateralization index (volume and HMOA) are displayed separately in ADHD participants and controls

The one-sample omnibus test revealed a statistically significant lateralization of the volumes of the SLF branches in the whole ADHD group (F(1) = 46.715, p = 0.000). Post hoc one-sample t-tests indicated that all three branches were significantly right-lateralized in ADHD participants. When the same analysis was conducted in controls, although there was a statistically significant overall lateralization (F(1) = 5.322, p = 0.032), post hoc one-sample t-tests showed that only the SLF III was significantly right-lateralized (Fig. 1, Table 1).

**Table 1:** Omnibus and post-hoc t-tests

Omnibus test	ADHD (N = 60)	Controls (N = 20)	Comparison
Overall lateralization volume	F(1,59) = 46.715 p < 0.**001**	F(1,19) = 5.322 p = 0.**032**	F(1,78) = 3.587 p = 0.062

Lateralization indices	1-sample t-tests		2-sample t-tests
	ADHD (N = 60)	Controls (N = 20)	Comparison
SLF 1 LI vol	t_(59)_ = .2.261 p = 0**.027**	t_(19)_ = − 1.273 p = 0.218	t_(78)_ = 2.271 p = 0.**026**
SLF 2 LI vol	t_(59)_ = 2.155 p = 0**.035**	t_(19)_ = 0.111 p = 0.913	t_(78)_ = 0.997 p = 0.322
SLF 3 LI vol	t_(59)_ = 10.977 p < 0**.001**	t_(19)_ = 5.913 p < 0**.001**	t_(78)_ = 0.557 p = 0.579

Overall lateralization HMOA	F(1,59) = 72.489 p < 0.**001**	F(1,19) = 13.010 p = 0.**002**	F(1,78) = 0.677 p = 0.413

Lateralization indices	1-sample t-tests		2-sample t-tests
	ADHD (N = 60)	Controls (N = 20)	Comparison
SLF 1 LI HMOA	t_(59)_ = -2.024 p = 0**.048**	t_(19)_ = -0.876 p = 0.392	t_(78)_ = 0.291 p = 0.772
SLF 2 LI HMOA	t_(59)_ = 7.859 p < 0**.001**	t_(19)_ = 5.244 p < 0**.001**	t_(78)_ = -0.328 p = 0.743
SLF 3 LI HMOA	t_(59)_ = 8.804 p < 0**.001**	t_(19)_ = 2.882 p = 0**.009**	t_(78)_ = 1.762 p = 0.082

This table reports the results of the omnibus tests for the overall lateralization of the volume and HMOA of the SLF branches; the post-hoc one-sample t-tests in ADHD participants and controls separately; and the post-hoc two-sample t-tests comparing ADHD participants and controls. Significant results are highlighted in bold.

Similarly, the one-sample omnibus test revealed a statistically significant lateralization of the HMOA, which reflects the microstructural organization of the SLF branches, in the ADHD group (F(1) = 72.489, p = 0.000). Post hoc one-sample t-tests indicated that all three branches were significantly lateralized in ADHD participants (the SLF I was left-lateralized, whereas the SLF II and III were right-lateralized). When the same analysis was conducted in controls, although there was a statistically significant overall lateralization (F(1) = 13.010, p = 0.002), post hoc t-tests showed that only the SLF II and III were significantly right-lateralized (Fig. 1, Table 1).

#### Group comparisons

As shown in Table 1, when we ran a two-sample omnibus test to compare the overall lateralization of the volumes of the SLF branches in ADHD participants and controls, we observed a trend towards a statistically significant difference between groups (F(1) = 3.587, p = 0.062). The sensitivity analysis comparing non-responders with controls indicated a statistically significant difference in the overall lateralization of the volumes of the SLF branches (F(1) = 4.249, p = 0.044). Post-hoc two sample t-tests showed a statistically significant group difference for the SLF I, which was right-lateralized only in non-responders (t_(52)_ = 3.058, p = 0.004), as also observed in [24]. In addition, no significant difference was observed for the lateralization of the volumes of the other SLF branches. Further, the sensitivity analysis comparing responders with controls did not yield significant results (F(1) = 1.665,p = 0.204).

When we ran the two-sample omnibus test to compare the overall lateralization of the HMOA of the SLF branches in ADHD participants and controls, we did not find a statistically significant difference between groups in both the main analysis (F(1) = 0.677, p = 0.413) (Table 1) and in the sensitivity analyses (F(1) = 0.085, p = 771 for non-responders and F(1) = 1.832, p = 0.183 for responders).

#### Correlations between SLF lateralization and clinico-neuropsychological profiles

Results of the correlation analyses investigating the association between the lateralization of the SLF branches and clinico-neuropsychological profiles in the whole ADHD sample are reported in Tables S1-S3. Results that survived Bonferroni correction for multiple comparisons (p ≤ 0.017) are summarized below.

**Fig. 2:**
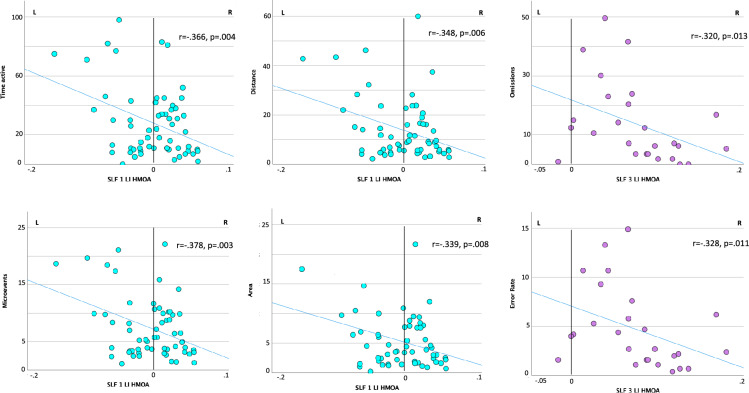
Correlations between SLF lateralization and Qb test parameters at baseline. At baseline, the SLF I LI HMOA was significantly negatively correlated with measures of hyperactivity whilst the SLF III LI HMOA was significantly negatively correlated with measures of inattention in the whole ADHD sample

**Fig. 3:**
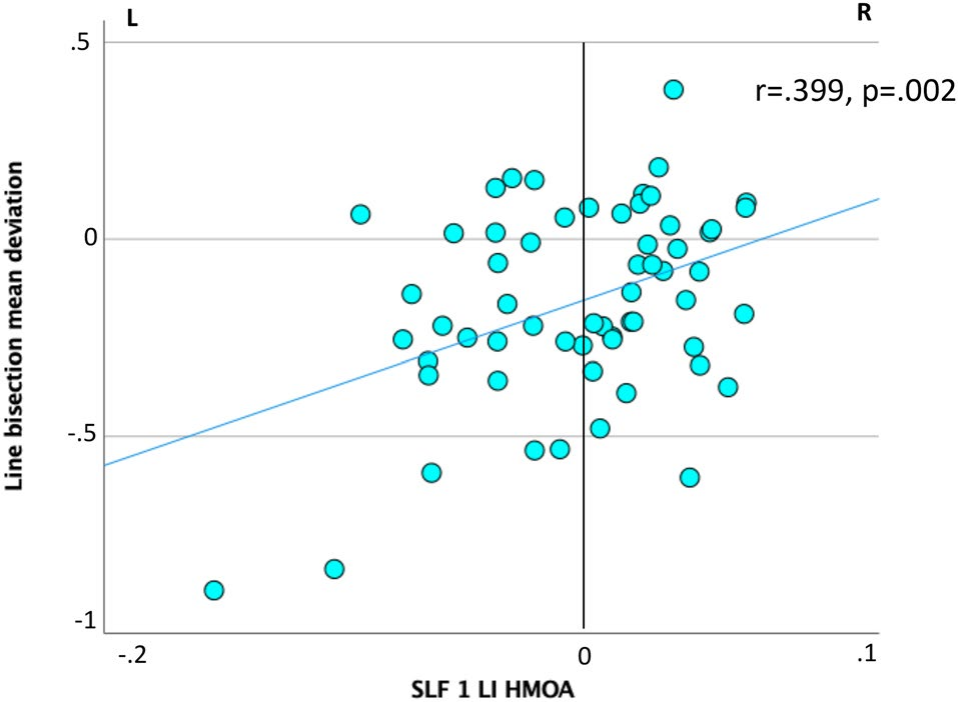
Correlations between SLF lateralization and line bisection. At baseline, a greater right lateralization of the SLF I HMOA was associated with more left deviation at the line bisection in the whole ADHD sample

Considering baseline clinico-neuropsychological profiles, the SLF I LI HMOA was significantly positively correlated with the mean deviation at the line bisection (r = 0.399, p = 0.002). It was also negatively correlated with time active (r = -0.366, p = 0.004), distance (r = -0.348, p = 0.006), area (r = − 0.339, p = 0.008), and microevents (r = -0.378, p = 0.003), which are Qb test parameters reflecting hyperactivity. Further, the SLF III LI HMOA significantly negatively correlated with omission errors (r = − 0.320, p = 0.013) and error rate (r = − 0.328, p = 0.011), which are Qb test parameters reflecting inattention. These results indicate that a greater right-lateralization of the SLF I HMOA was significantly associated with a more evident left deviation at the line bisection, and with less pre-treatment hyperactivity as measured by the Qb test. Further, a greater right-lateralization of the SLF III HMOA was significantly associated with fewer inattention-related errors at the Qb test (Fig. 2–3). Finally, significant correlations were observed with symptom and neuropsychological improvement under an acute dose of MPH or at follow-up, but these did not survive Bonferroni correction (Tables S2–S3).

#### Secondary analyses

We observed a significant negative correlation between the SLF II LI HMOA and total IQ (r = − 0.287, p = 0.026), however this did not survive correction for multiple comparisons (p ≥ 0.017) (Table S4). When we correlated corresponding lateralization indices based on volume and HMOA, we observed positive significant correlations between lateralization parameters of the SLF II (r = 0.258, p = 0.047) and SLF III (r = 0.473, p < 0.001). The latter survived correction for multiple comparisons (p ≤ 0.017) (Table S5).

Group comparisons based on lateralization composite scores indicated a trend towards a difference in overall lateralization (F (1,78) = 3.501, p = 0.065), primarily driven by the SLF I (Table S6). No correlation between lateralization composite scores and clinico-neuropsychological variables survived correction for multiple comparisons (all p ≥ 0.017) (Tables S7, S8, S9).

## Discussion

### Summary of findings

This study showed, for the first time, that all three SLF branches were significantly lateralized in adults with ADHD, but not in neurotypical controls. These results partly confirm prior findings in neurotypical adults, i.e. the right-lateralization of the SLF III volume [14] and SLF II HMOA [39], although we also observed a right-lateralization of the SLF III HMOA. Further, although ADHD participants and controls did not significantly differ in their pattern of lateralization when directly compared, we observed that asymmetry of the SLF branches was associated with variation in neuropsychological performance in the ADHD group. For instance, the lateralization of the SLF I HMOA was associated with performance at the line bisection, not that of the SLF II volume as previously reported in controls [14]. In addition, the SLF I HMOA was significantly left-lateralized in ADHD participants (but bilateral in controls), and an increased left-lateralization was associated with higher levels of hyperactivity as measured by the Qb test at baseline. Finally, the SLF III HMOA was significantly right-lateralized in ADHD participants (as in controls) and an increased right-lateralization was associated with lower levels of inattention as measured by the Qb test at baseline. Overall, these findings suggest that asymmetry of the SLF branches contributes to variation in neuropsychological profiles in adults with ADHD.

### Interpretation of findings

A failure to develop a typical pattern of asymmetry has been suggested to underpin a range of neurodevelopmental conditions, from dyslexia to autism and schizophrenia [40, 41]. For example, dyslexia has been associated with altered asymmetries within language networks [42, 43], whereas multiple tract alterations have been observed in autism and schizophrenia [41, 44]. Notably, altered patterns of asymmetry have been reported to also correlate with symptom severity and chronicity [45]. Regarding ADHD, several prior imaging studies have suggested a complex pattern of altered laterality [17, 46, 47]. Unfortunately, so far, only a DWI study investigated interhemispheric differences in the microstructural organization of the SLF in ADHD, but was in children, focused on the dorsal branch, and did not investigate its clinico-neuropsychological correlates [17]. Nevertheless, two recent studies combining tractography with network analysis showed that an altered asymmetric regional efficiency, predominantly in fronto-striatal connections, was associated with symptom severity and cognitive performance in adults with ADHD [48, 49]. Taken together, these and our findings suggest that brain network asymmetry, including that of the SLF branches, may contribute to ADHD pathophysiology. Longitudinal studies on the developmental trajectories of network asymmetries are needed to clarify their exact role and possible differences between pediatric and adult samples. Considering age-related differences, it is worth noting that a recent meta-analysis/meta-regression of TBSS studies reported that the most significant case–control differences, which were observed in the body and splenium of the corpus callosum, were negatively associated with age and did not survive in the pediatric meta-analysis [50]. Similarly, a study including 120 children and adults with ADHD with 23 matched controls observed significantly reduced FA in several brain regions in adults with ADHD as compared to controls but no group differences in treatment-naïve children [18]. These findings contrast prior structural MRI meta- and mega-analyses reporting significant ADHD vs control differences in volumetric and morphometric measures in children but not in adults with ADHD, thus indicating a lessening of brain alterations with growing age [7, 51]. This inconsistency might be related to the fact that, whilst grey matter measures reach their peak in childhood and then decrease [52], white matter development reaches its peak between 21 and 29 years [53, 54].Therefore, a delayed white matter maturation in ADHD would result in more evident case–control differences in older individuals [18, 50]. Finally, there is evidence that individual variation in white matter developmental trajectories may relate to the variable outcome of ADHD in adulthood. In fact, white matter development in cortical areas subserving cognitive/emotional skills continues up to early adulthood, coinciding with the typical age of ADHD symptom improvement/remission [55, 56]. Follow-up studies based on NeuroIMAGE samples showed that lower FA at follow-up in the region where the left corticospinal tract crosses the SLF was associated with improvement in combined ADHD and hyperactivity-impulsivity symptoms in late adolescence-early adulthood [56, 57]. However, a later study from the same group following-up participants up to 34 years of age identified significant associations only between higher fiber density and fiber cross-section in the left corticospinal tract and symptom improvement [58]. Therefore, future studies may want to investigate how developmental trajectories of white matter tract asymmetry may relate to symptom persistence or remission.

The potential pathophysiological role of an asymmetry of attentive networks in ADHD is supported by the results of our correlation analyses. For instance, we found an association between the lateralization of the microstructural organization of the SLF I and measures of attention and hyperactivity in the whole ADHD sample. These results are in line with the previously reported role of the dorsal branch of the SLF in supporting brain functions such as voluntary-oriented attention, working memory, and motor control [14, 20, 23]. Prior studies also reported that reduced microstructural organization of the right SLF was associated with increased reaction time variability during sustained attention in adult ADHD [59] and with poorer hand-motor coordination in children with ADHD [60]. Although these studies either investigated the SLF as a singles bundle or focused on children, thus limiting the comparisons that can be made, they suggest (in line with our findings) that asymmetry of SLF microstructural organization is particularly relevant to neuropsychological functions affected in ADHD. In contrast to our results, prior studies also reported an association between the microstructural organization of the SLF and symptom severity in ADHD [61, 62]. Inconsistencies may be related to the fact that these prior studies investigated the SLF as a single bundle, mainly in children or adolescents, and did not specifically analyze interhemispheric asymmetry.

The results of the line bisection also support our hypothesis of a different functional organization of fronto-parietal attentive networks leading to ADHD pathophysiology. At baseline, we noted a small left deviation at a group level. This phenomenon, known as ‘pseudoneglect effect’, has been previously reported in neurotypical individuals and related to the dominance of the right hemisphere in visuospatial attentive processing [63]. However, we observed a different relationship between this left deviation and the pattern of lateralization of the SLF branches in our ADHD participants, as compared to what was previously reported in neurotypical adults. Specifically, we observed that a greater left deviation was associated with a greater right lateralization of the SLF I HMOA in our ADHD group, whilst a prior study reported that it was associated with a greater right lateralization of the SLF II volume in neurotypicals [14]. Similarly, a recent study showed that a right lateralization of the SLF II HMOA was associated with more left forward spatial bias in neurotypical adults [39]. These findings suggest that the lateralization of the SLF I may be more relevant to optimal performance at the line bisection in individuals with ADHD, and especially its microstructure. Further, they support the suggestion that microstructural (HMOA) and macrostructural organization (volume) of the SLF may differentially contribute to individual variability in anatomical lateralization and neuropsychological profiles. In line with this suggestion, a prior study reported that variation in the right SLF II and III HMOA was associated with working memory, whilst both right SLF II HMOA and volume were associated with spatial bias in neurotypicals [39]. In agreement, we observed limited correlations between corresponding SLF lateralization indices based on volume and HMOA and, when we calculated an average lateralization, we could not observe the significant associations identified when studying these indices separately. Taken together, these findings suggest that variation in micro and macrostructural asymmetry of fronto-parietal networks may be differentially associated with variability in cognitive performance in ADHD and neurotypical individuals.

### Underlying biological mechanisms

Unfortunately, tractography does not allow direct investigation of the biological mechanisms underlying anatomical asymmetry. A lateralized tract volume may reflect different factors, including the number of axons, their diameter, or degree of myelination, which affect conduction speed [37, 38]. Similarly, an asymmetrical microstructural organization may reflect changes in diffusivity due to axonal loss, altered myelination processes, or differences in fiber diameter [36], which ultimately may hinder neuronal function. It is also not known how asymmetry may relate to neuropsychological performance. Nevertheless, it is possible to speculate that, as the SLF I in neurotypical subjects is a bilateral tract that supports cognitive functions such as the voluntary control of attention and behavior, an asymmetrical representation of the SLF I may condition an imbalance between hemispheric contributions during performance of higher cognitive and motor functions implicated in ADHD. Conversely, a right-lateralization of the SLF III (as in controls) may support more effective stimulus-driven attention processes [23].

### Strengths, limitations, and future directions

This study has several strengths, such as the longitudinal design, the advanced tractography method, and the relatively large sample of adults with ADHD, which allowed us to separate treatment responders and non-responders. Limitations should also be considered. First, we included only males because ADHD is more commonly diagnosed in males [26], and there is preliminary evidence of sex-related differences in anatomical connectivity [27–30] and treatment response [31–33]. However, it is not known how these differences may interplay, thus, to avoid sex-related confounding, we restricted the analysis to males. Future studies should test whether the observed findings are generalizable to the female ADHD population. Similarly, we included only ADHD participants without current comorbid conditions because neuroanatomical differences have been reported between individuals with/without comorbidities [9]. Nevertheless, our results should be extended to clinical samples also including subjects with comorbidities. Further, we included a small proportion of individuals previously treated with ADHD medication. However, most participants were medication-naïve and previous studies have excluded a ‘normalizing’ effect of stimulants on brain structure [64]. Finally, we included a relatively small sample of controls. This is because the original trial this work originated from aimed at identifying ‘predictors’ of treatment response in a cohort of ADHD participants, and controls were included only for secondary analyses to support interpretation of primary findings. Nevertheless, our results in neurotypicals mostly confirmed findings from prior studies.

In conclusion, our and prior findings indicate that the pattern of lateralization of the SLF branches plays a role in neurocognitive performance in both ADHD and neurotypical adults; although we showed that altered asymmetry, perhaps especially of the dorsal branch (SLF I), contributes to ADHD pathophysiology. Ultimately, these results provide new insight into brain mechanisms underpinning ADHD-related neurocognitive deficits and may help guide the optimization of targeted treatment strategies, such as cognitive training.

## Supplementary Information

Below is the link to the electronic supplementary material.

Supplementary file1 (DOCX 70 KB)
