# Supplementary material for: Asymmetry of attentive networks contributes to adult Attention-deficit/hyperactivity disorder (ADHD) pathophysiology
Source: Eur Arch Psychiatry Clin Neurosci. 2024 Nov 2;275(7):1885–95. doi: 10.1007/s00406-024-01927-4 (PMC12589327; doi:10.1007/s00406-024-01927-4)
Supplement: Supplementary file 1 — Supplementary file1 (DOCX 70 KB) [file 406_2024_1927_MOESM1_ESM.docx]

**SUPPLEMENTARY MATERIAL**

**TABLE OF CONTENT**

**Clinical and behavioural measures Page 2**

**Treatment response Page 3**

Table S1. Correlations between lateralization and clinico-neuropsychological

profiles at baseline Page 4

Table S2. Correlations between lateralization and change in clinico-

neuropsychological profiles under an acute dose of methylphenidate (MPH) Page 5

Table S3. Correlations between lateralization and change in clinico-

neuropsychological profiles at follow-up Page 6

Table S4. Correlations between SLF lateralization indices, handedness, and total IQ. Page 7

Table S5. Correlations between lateralization indices based on volume and HMOA. Page 7

Table S6. Omnibus and post-hoc t-tests for lateralization composite scores. Page 8

Table S7. Correlations between lateralization composite scores and clinico-

neuropsychological profiles at baseline. Page 8

Table S8. Correlations between lateralization composite scores and change in clinico-

neuropsychological profiles under an acute dose of methylphenidate (MPH). Page 9

Table S9. Correlations between lateralization composite scores and change in clinico-

neuropsychological profiles at follow-up. Page 10

**Abbreviations Page 11**

**References Page 11**

**CLINICAL AND BEHAVIOURAL MEASURES**

The full research protocol has been previously described in [1], which reported the results of the analysis investigating diffusion imaging ‘predictors’ of treatment response in ADHD.

At baseline, **handedness** was measured with a modified version of the Edinburgh Handedness Inventory (EHI)[2], and    intelligent quotient (**IQ**) with the Wechsler Abbreviated Scale of Intelligence (WASI)[3].

**Clinical symptoms** were measured at each time point (baseline, single dose of methylphenidate (MPH), and two-month follow-up) using the Barkley Adult ADHD Rating Scale-IV (BAARS-IV)[4], which provided three scores (ADHD Total score, ADHD Inattention, and ADHD Hyperactivity-Impulsivity).

Behavioral tests included the Line bisection [5] and the Quantitative behavior (Qb) test (<https://www.qbtech.com>). In the **Line bisection** test, subjects were requested to mark the center of 20 horizontal lines with a pencil, alternatively using their right or left hand, and the deviation of the bisection mark from the true center was measured as an indicator of asymmetrical visuospatial attention processing. Participants completed the line bisection test under placebo and under an acute dose of MPH, and we considered the mean deviation with both hands under each condition. We selected this test as a prior study reported an association between the pattern of lateralization of the SLF branches and performance at the line bisection in neurotypical adults [6].

The **Qb test** is a computer-based test that measures core ADHD symptoms through a continuous performance task (CPT) and infrared monitoring of an individual’s movements. We selected this test as it was approved by the Food and Drug Administration to aid treatment response monitoring in ADHD participants [7]. The Qb test provides three summary scores (Qb activity, Qb impulsivity and Qb inattention), which reflect ADHD symptom domains. In this study we considered the three Qb scores but also the underlying individual parameters. Qb Activity (QbAct) includes parameters measured by the motion-capturing device, such as distance and area covered by the marker, time active (i.e., time in percent one moves more than 1cm/sec), and microevents (i.e., how many times one moves more than 1mm). Qb Inattention (QbIna) and Qb Impulsivity (QbImp) include parameters derived from the CPT. Specifically, QbIna includes omission errors, reaction time (RT), and RT variability (i.e., the standard deviation of RT); and QbImp includes commission errors and normalized commission errors (i.e., the ratio of commission errors to the number of corrected responses). Participants completed the Qb test at the three time points (baseline, acute MPH and follow-up). Behavioral test results were extracted by a researcher blind to the clinical profiles of participants.

**TREATMENT RESPONSE**

Individuals with ADHD were classified as responders or non-responders to MPH at two months using a data-driven approach as previously described [1, 8]. We did not use a symptom improvement cut-off to separate responders and non-responders because, although commonly used in clinical practice and trials, a classification based on an arbitrary cut-off might not reflect a biological distinction among participants or apply to a specific sample. For this reason, we employed a data-driven approach, multivariate k-mean clustering [1, 8]. This is a common algorithm to cluster multivariate data and defines whether participants belong to different groups based on their multiple characteristics. The allocation to a group is made so to minimize the squared error between the empirical mean and the cluster observations [9]. The segregation of our sample into responders and non-responders was based on their improvement on inattentive or hyperactive-impulsive symptoms and three Qb scores at follow-up as compared to baseline. Further details can be found in [1].

**Table S1.** **Correlations between lateralization and clinico-neuropsychological profiles at baseline.** Asterisks indicate significant correlations. Those that survived Bonferroni correction for multiple comparisons (p<=0.017) are highlighted in bold.

|  | **SLF 1 LI Vol** | **SLF 2 LI Vol** | **SLF 3 LI Vol** | **SLF 1 LI HMOA** | **SLF 2 LI HMOA** | **SLF 3 LI HMOA** |
| --- | --- | --- | --- | --- | --- | --- |
| **BASELINE** | r (p value) | r (p value) | r (p value) | r (p value) | r (p value) | r (p value) |
| **Whole ADHD sample (N=60)** | | | | | | |
| BAARS total | -0.004 | -0.008 | 0.129 | -0.072 | 0.052 | -0.004 |
|  | 0.973 | 0.953 | 0.326 | 0.587 | 0.693 | 0.976 |
| BAARS Ina | 0.003 | -0.098 | 0.072 | -0.162 | 0.036 | 0.113 |
|  | 0.984 | 0.455 | 0.585 | 0.217 | 0.786 | 0.388 |
| BAARS Hyp/imp | -0.087 | 0.013 | 0.110 | -0.096 | 0.105 | -0.117 |
|  | 0.511 | 0.922 | 0.402 | 0.464 | 0.426 | 0.375 |
| LINE mean | -0.121 | 0.059 | 0.040 | **.399^**^** | -0.114 | 0.100 |
|  | 0.355 | 0.657 | 0.760 | **0.002** | 0.386 | 0.448 |
| Qb Act | 0.204 | -0.058 | -0.001 | -.277^*^ | -0.029 | -0.172 |
|  | 0.117 | 0.661 | 0.995 | 0.032 | 0.824 | 0.189 |
| QB Imp | 0.134 | -0.155 | 0.001 | -0.060 | -0.212 | -0.172 |
|  | 0.307 | 0.238 | 0.992 | 0.648 | 0.104 | 0.190 |
| QB Ina | 0.033 | 0.054 | -0.152 | -.304^*^ | 0.066 | -0.205 |
|  | 0.801 | 0.680 | 0.245 | 0.018 | 0.616 | 0.116 |
| Time active | 0.134 | -0.131 | 0.028 | **-.366^**^** | -0.067 | -0.165 |
|  | 0.308 | 0.319 | 0.833 | **0.004** | 0.613 | 0.208 |
| Distance | 0.152 | -0.200 | 0.026 | **-.348^**^** | -0.072 | -0.225 |
|  | 0.247 | 0.125 | 0.844 | **0.006** | 0.582 | 0.084 |
| Area | 0.130 | -0.149 | 0.009 | **-.339^**^** | -0.105 | -0.250 |
|  | 0.322 | 0.256 | 0.948 | **0.008** | 0.423 | 0.054 |
| Microevents | 0.155 | -0.148 | 0.038 | **-.378^**^** | -0.051 | -0.189 |
|  | 0.236 | 0.259 | 0.776 | **0.003** | 0.699 | 0.147 |
| Omissions | .267^*^ | -0.040 | -0.199 | -.255^*^ | -0.166 | **-.320^*^** |
|  | 0.040 | 0.763 | 0.128 | 0.049 | 0.206 | **0.013** |
| Commissions | 0.043 | -0.106 | -0.024 | -0.092 | -0.216 | -0.189 |
|  | 0.743 | 0.422 | 0.854 | 0.486 | 0.098 | 0.149 |
| Error rate | 0.239 | -0.064 | -0.180 | -0.245 | -0.202 | **-.328^*^** |
|  | 0.066 | 0.627 | 0.169 | 0.060 | 0.121 | **0.011** |
| RT var | 0.139 | 0.101 | -0.086 | -0.229 | -0.093 | -0.226 |
|  | 0.290 | 0.445 | 0.516 | 0.079 | 0.479 | 0.083 |

**Table S2.** **Correlations between lateralization and change in clinico-neuropsychological profiles under an acute dose of methylphenidate (MPH).** Asterisks indicate significant correlations. Those that survived Bonferroni correction for multiple comparisons (p<=0.017) are highlighted in bold. Of note, 15 subjects did not complete the Barkley under an acute dose of MPH.

|  | **SLF 1 LI Vol** | **SLF 2 LI Vol** | **SLF 3 LI Vol** | **SLF 1 LI HMOA** | **SLF 2 LI HMOA** | **SLF 3 LI HMOA** |
| --- | --- | --- | --- | --- | --- | --- |
| **CHANGES UNDER ACUTE DOSE** | r (p value) | r (p value) | r (p value) | r (p value) | r (p value) | r (p value) |
| **Whole ADHD sample (N=60)** | | | | | | |
| BAARS total | 0.036 | 0.342^*^ | 0.048 | -0.026 | 0.010 | -0.295^*^ |
|  | 0.813 | 0.022 | 0.753 | 0.864 | 0.950 | 0.049 |
| BAARS Ina | -0.083 | 0.314^*^ | -0.063 | -0.206 | -0.094 | -0.295^*^ |
|  | 0.588 | 0.036 | 0.683 | 0.174 | 0.538 | 0.049 |
| BAARS Hyp/imp | 0.086 | 0.212 | 0.142 | -0.023 | 0.034 | -0.185 |
|  | 0.575 | 0.162 | 0.352 | 0.883 | 0.823 | 0.224 |
| LINE mean | -.287^*^ | 0.069 | -0.211 | 0.178 | 0.098 | -0.140 |
|  | 0.026 | 0.600 | 0.105 | 0.172 | 0.458 | 0.286 |
| Qb Act | -0.061 | 0.232 | 0.025 | -0.108 | -0.074 | -0.107 |
|  | 0.645 | 0.075 | 0.849 | 0.413 | 0.574 | 0.417 |
| QB Imp | -0.152 | -0.023 | -0.070 | 0.008 | -0.128 | 0.061 |
|  | 0.246 | 0.860 | 0.594 | 0.953 | 0.331 | 0.646 |
| QB Ina | -0.078 | 0.052 | -0.123 | -0.263^*^ | -0.088 | -0.122 |
|  | 0.553 | 0.695 | 0.351 | 0.042 | 0.502 | 0.351 |
| Time active | 0.026 | 0.207 | 0.153 | -0.202 | -0.104 | -0.043 |
|  | 0.841 | 0.112 | 0.242 | 0.121 | 0.429 | 0.745 |
| Distance | 0.075 | 0.272^*^ | 0.136 | -0.258^*^ | 0.037 | -0.026 |
|  | 0.567 | 0.035 | 0.299 | 0.047 | 0.781 | 0.842 |
| Area | -0.044 | 0.264^*^ | 0.039 | -0.170 | 0.015 | -0.103 |
|  | 0.737 | 0.042 | 0.765 | 0.193 | 0.908 | 0.434 |
| Microevents | 0.035 | .280^*^ | 0.138 | -0.198 | -0.028 | -0.030 |
|  | 0.792 | 0.030 | 0.293 | 0.129 | 0.830 | 0.820 |
| Omissions | 0.125 | -0.044 | -0.182 | -.261^*^ | -0.202 | -0.189 |
|  | 0.342 | 0.739 | 0.163 | 0.044 | 0.122 | 0.147 |
| Commissions | -0.185 | 0.028 | 0.016 | 0.052 | -0.067 | 0.080 |
|  | 0.156 | 0.835 | 0.901 | 0.692 | 0.609 | 0.543 |
| Error rate | 0.005 | 0.131 | -0.074 | 0.062 | 0.116 | -0.052 |
|  | 0.969 | 0.319 | 0.572 | 0.637 | 0.377 | 0.691 |
| RT var | -0.096 | 0.092 | -0.145 | -0.117 | -0.129 | -0.052 |
|  | 0.464 | 0.484 | 0.268 | 0.375 | 0.327 | 0.693 |

**Table S3 Correlations between lateralization and change in clinico-neuropsychological profiles at follow-up.** Asterisks indicate significant correlations. Those that survived Bonferroni correction for multiple comparisons (p<=0.017) are highlighted in bold.

|  | **SLF 1 LI Vol** | **SLF 2 LI Vol** | **SLF 3 LI Vol** | **SLF 1 LI HMOA** | **SLF 2 LI HMOA** | **SLF 3 LI HMOA** |
| --- | --- | --- | --- | --- | --- | --- |
| **CHANGES AT FOLLOW-UP** | r (p value) | r (p value) | r (p value) | r (p value) | r (p value) | r (p value) |
| **Whole ADHD sample (N=60)** | | | | | | |
| BAARS total | -0.136 | 0.191 | -0.155 | 0.061 | 0.025 | 0.042 |
|  | 0.302 | 0.144 | 0.237 | 0.641 | 0.850 | 0.752 |
| BAARS Ina | -0.170 | 0.127 | -0.178 | 0.119 | 0.228 | 0.099 |
|  | 0.195 | 0.333 | 0.174 | 0.366 | 0.079 | 0.454 |
| BAARS Hyp/imp | -0.156 | 0.229 | -0.126 | -0.047 | -0.013 | -0.059 |
|  | 0.233 | 0.079 | 0.338 | 0.724 | 0.922 | 0.657 |
| Qb Act | 0.100 | 0.102 | -0.178 | -0.059 | 0.142 | -0.094 |
|  | 0.447 | 0.440 | 0.174 | 0.656 | 0.279 | 0.473 |
| QB Imp | -0.109 | 0.016 | -0.122 | 0.094 | -0.092 | 0.083 |
|  | 0.408 | 0.905 | 0.355 | 0.473 | 0.486 | 0.529 |
| QB Ina | -0.005 | 0.074 | -0.186 | -0.170 | -0.045 | -0.211 |
|  | 0.968 | 0.573 | 0.154 | 0.195 | 0.731 | 0.105 |
| Time active | 0.112 | -0.053 | -0.030 | -0.232 | 0.094 | -0.132 |
|  | 0.396 | 0.690 | 0.822 | 0.075 | 0.474 | 0.315 |
| Distance | 0.119 | -0.127 | 0.015 | -.267^*^ | 0.080 | -0.184 |
|  | 0.366 | 0.333 | 0.907 | 0.039 | 0.546 | 0.160 |
| Area | 0.081 | -0.050 | -0.019 | -.281^*^ | 0.063 | -0.223 |
|  | 0.537 | 0.703 | 0.884 | 0.030 | 0.632 | 0.087 |
| Microevents | 0.124 | -0.044 | 0.001 | -.278^*^ | 0.128 | -0.143 |
|  | 0.345 | 0.739 | 0.996 | 0.032 | 0.331 | 0.277 |
| Omissions | 0.114 | 0.084 | -0.248 | -0.230 | -0.096 | -0.233 |
|  | 0.387 | 0.522 | 0.056 | 0.077 | 0.467 | 0.073 |
| Commissions | -0.160 | 0.026 | -0.078 | 0.087 | -0.107 | -0.008 |
|  | 0.222 | 0.844 | 0.551 | 0.511 | 0.418 | 0.950 |
| Error rate | 0.074 | 0.085 | -.261^*^ | -0.200 | -0.117 | -0.230 |
|  | 0.575 | 0.518 | 0.044 | 0.125 | 0.374 | 0.077 |
| RT var | 0.110 | 0.108 | -0.086 | -0.042 | -0.148 | -0.160 |
|  | 0.404 | 0.413 | 0.513 | 0.748 | 0.260 | 0.222 |

**Table S4. Correlations between SLF lateralization indices, handedness, and total IQ.** We observed a significant negative correlation between the SLF 2 LI HMOA and IQ, however this did not survive correction for multiple comparisons (p>=0.017).

|  | **Handedness** | **Total IQ** |
| --- | --- | --- |
|  | **r**  **(p-value)** | **r**  **(p-value)** |
| **SLF 1 LI Vol** | -0.028 | -0.164 |
|  | 0.833 | 0.212 |
| **SLF 2 LI Vol** | -0.060 | -0.103 |
|  | 0.647 | 0.432 |
| **SLF 3 LI Vol** | -0.180 | -0.058 |
|  | 0.168 | 0.658 |
| **SLF 1 LI HMOA** | 0.079 | -0.147 |
|  | 0.546 | 0.261 |
| **SLF 2 LI HMOA** | -0.097 | *-.287^*^* |
|  | 0.459 | *0.026* |
| **SLF 3 LI HMOA** | -0.174 | -0.058 |
|  | 0.183 | 0.661 |

**Table S5. Correlations between corresponding lateralization indices based on volume and HMOA**. We observed positive significant correlations between lateralization parameters of the SLF 2 and SLF 3. The latter survived correction for multiple comparisons (p<=0.017).

|  | **SLF 1 LI HMOA** | **SLF 2 LI HMOA** | **SLF 3 LI HMOA** |
| --- | --- | --- | --- |
|  | **r**  **(p-value)** | **r**  **(p-value)** | **r**  **(p-value)** |
| **SLF 1 LI Vol** | 0.194 |  |  |
|  | 0.138 |  |  |
| **SLF 2 LI Vol** |  | *.258^*^* |  |
|  |  | *0.047* |  |
| **SLF 3 LI Vol** |  |  | **.473^**^** |
|  |  |  | **0.000** |

**Table S6.** **Omnibus and post-hoc t-tests for lateralization composite scores.** Group comparisons based on lateralization composite scores indicated a trend towards a difference in overall lateralization, primarily driven by the SLF 1. Significant results are highlighted in bold.

| **OMNIBUS TEST** | **COMPARISON**  **ADHD vs controls** |
| --- | --- |
| **Overall lateralization** | F(1,78)=3.501  p=.065 |
|  | **2-sample t-test** |
| **SLF 1 LI composite** | t(78)=-2.210, p=**.030** |
| **SLF 2 LI composite** | t(78)=-.800, p=.426 |
| **SLF 3 LI composite** | t(78)=-.975, p=.333 |

**Table S7.** **Correlations between lateralization composite scores and clinico-neuropsychological profiles at baseline.** Asterisks indicate significant correlations. None survived Bonferroni correction for multiple comparisons (p>=0.017).

|  | **SLF 1 LI composite** | **SLF 2 LI composite** | **SLF 3 LI composite** |
| --- | --- | --- | --- |
| **BASELINE** | r (p value) | r (p value) | r (p value) |
| BAARS total | -0.021 | 0.007 | 0.106 |
|  | 0.872 | 0.955 | 0.421 |
| BAARS Ina | -0.036 | -0.078 | 0.093 |
|  | 0.782 | 0.555 | 0.482 |
| BAARS Hyp/imp | -0.103 | 0.041 | 0.058 |
|  | 0.433 | 0.759 | 0.661 |
| LINE mean | -0.017 | 0.021 | 0.062 |
|  | 0.9 | 0.876 | 0.636 |
| Qb Act | 0.110 | -0.197 | -0.048 |
|  | 0.404 | 0.132 | 0.713 |
| QB Imp | -0.042 | 0.067 | -0.186 |
|  | 0.748 | 0.612 | 0.156 |
| QB Ina | 0.035 | -0.174 | 0.119 |
|  | 0.792 | 0.183 | 0.365 |
| Time active | 0.036 | -0.135 | -0.025 |
|  | 0.785 | 0.303 | 0.852 |
| Distance | 0.057 | -0.199 | -0.044 |
|  | 0.666 | 0.128 | 0.741 |
| Area | 0.039 | -0.162 | -0.065 |
|  | 0.768 | 0.216 | 0.621 |
| Microevents | 0.053 | -0.146 | -0.024 |
|  | 0.688 | 0.265 | 0.858 |
| Omissions | 0.185 | -0.081 | -.258^*^ |
|  | 0.156 | 0.536 | 0.047 |
| Commissions | 0.018 | -0.154 | -0.075 |
|  | 0.891 | 0.240 | 0.571 |
| Error rate | 0.163 | -0.113 | -0.244 |
|  | 0.215 | 0.389 | 0.060 |
| RT var | 0.074 | 0.064 | -0.136 |
|  | 0.576 | 0.628 | 0.299 |

**Table S8.** **Correlations between lateralization composite scores and change in clinico-neuropsychological profiles under an acute dose of methylphenidate (MPH).** Asterisks indicate significant correlations. None survived Bonferroni correction for multiple comparisons (p>=0.017).

|  | **SLF 1 LI composite** | **SLF 2 LI composite** | **SLF 3 LI composite** |
| --- | --- | --- | --- |
| **CHANGES UNDER ACUTE DOSE** | r (p value) | r (p value) | r (p value) |
| BAARS total | 0.027 | .306^*^ | -0.047 |
|  | 0.860 | 0.041 | 0.757 |
| BAARS Ina | -0.122 | 0.251 | -0.138 |
|  | 0.425 | 0.097 | 0.365 |
| BAARS Hyp/imp | 0.073 | 0.198 | 0.062 |
|  | 0.632 | 0.192 | 0.687 |
| LINE mean | 0.148 | -0.046 | 0.214 |
|  | 0.259 | 0.725 | 0.101 |
| Qb Act | -0.082 | 0.186 | -0.010 |
|  | 0.534 | 0.155 | 0.939 |
| QB Imp | -0.139 | -0.056 | -0.041 |
|  | 0.290 | 0.669 | 0.757 |
| QB Ina | -0.135 | 0.022 | -0.137 |
|  | 0.302 | 0.870 | 0.296 |
| Time active | -0.024 | 0.156 | 0.115 |
|  | 0.855 | 0.234 | 0.382 |
| Distance | 0.008 | 0.253 | 0.106 |
|  | 0.952 | 0.051 | 0.422 |
| Area | -0.082 | 0.239 | 0.003 |
|  | 0.534 | 0.065 | 0.982 |
| Microevents | -0.015 | 0.242 | 0.106 |
|  | 0.907 | 0.062 | 0.420 |
| Omissions | 0.053 | -0.095 | -0.206 |
|  | 0.688 | 0.470 | 0.114 |
| Commissions | -0.159 | 0.006 | 0.037 |
|  | 0.225 | 0.965 | 0.780 |
| Error rate | 0.020 | 0.149 | -0.077 |
|  | 0.881 | 0.256 | 0.559 |
| RT var | -0.117 | 0.046 | -0.136 |
|  | 0.373 | 0.725 | 0.301 |

**Table S9. Correlations between lateralization composite scores and change in clinico-neuropsychological profiles at follow-up.** Asterisks indicate significant correlations. None survived Bonferroni correction for multiple comparisons (p>=0.017).

|  | **SLF 1 LI composite** | **SLF 2 LI composite** | **SLF 3 LI composite** |
| --- | --- | --- | --- |
| **CHANGES AT FOLLOW-UP** | r (p value) | r (p value) | r (p value) |
| BAARS total | -0.111 | 0.177 | -0.117 |
|  | 0.400 | 0.176 | 0.374 |
| BAARS Ina | -0.129 | 0.177 | -0.119 |
|  | 0.328 | 0.177 | 0.365 |
| BAARS Hyp/imp | -0.156 | 0.200 | -0.122 |
|  | 0.234 | 0.125 | 0.355 |
| Qb Act | 0.079 | 0.130 | -0.175 |
|  | 0.551 | 0.322 | 0.181 |
| QB Imp | -0.078 | -0.011 | -0.077 |
|  | 0.553 | 0.931 | 0.559 |
| QB Ina | -0.046 | 0.054 | -0.216 |
|  | 0.729 | 0.684 | 0.098 |
| Time active | 0.048 | -0.021 | -0.063 |
|  | 0.718 | 0.875 | 0.634 |
| Distance | 0.046 | -0.091 | -0.040 |
|  | 0.727 | 0.488 | 0.760 |
| Area | 0.008 | -0.027 | -0.080 |
|  | 0.953 | 0.836 | 0.541 |
| Microevents | 0.048 | -0.004 | -0.041 |
|  | 0.715 | 0.978 | 0.757 |
| Omissions | 0.050 | 0.049 | -.273^*^ |
|  | 0.704 | 0.713 | 0.035 |
| Commissions | -0.127 | -0.006 | -0.068 |
|  | 0.332 | 0.961 | 0.608 |
| Error rate | 0.020 | 0.044 | -.283^*^ |
|  | 0.878 | 0.741 | 0.029 |
| RT var | 0.091 | 0.055 | -0.118 |
|  | 0.488 | 0.676 | 0.370 |

**ABBREVIATIONS**

BAARS total: Barkley Adult ADHD Rating Scale-IV Total score

BAARS Ina: Barkley Adult ADHD Rating Scale-IV Inattentive score

BAARS Hyp/imp: Barkley Adult ADHD Rating Scale-IV Hyperactivity/impulsivity score

IQ: Intelligent quotient

Line mean: Line bisection, mean deviation

Qb Act: Qb Activity

Qb Imp: Qb Impulsivity

Qb Ina: Qb Inattention

RT var: Reaction Time variability

**REFERENCES**

1. Parlatini, V., et al., *Poor response to methylphenidate is associated with a smaller dorsal attentive network in adult Attention-Deficit/Hyperactivity Disorder (ADHD).* Transl Psychiatry, 2023. **13**(1): p. 303.

2. Oldfield, R.C., *The assessment and analysis of handedness: the Edinburgh inventory.* Neuropsychologia, 1971. **9**(1): p. 97-113.

3. Wechsler, D., *Wechsler abbreviated scale of intelligence.* 1999.

4. Barkley, R.A., *Barkley Adult ADHD Rating Scale-IV (BAARS-IV)*. 2011, New York: Guilford Press. ix, 150 p.

5. Schenkenberg, T., D.C. Bradford, and E.T. Ajax, *Line bisection and unilateral visual neglect in patients with neurologic impairment.* Neurology, 1980. **30**(5): p. 509-17.

6. Thiebaut de Schotten, M., et al., *A lateralized brain network for visuospatial attention.* Nat Neurosci, 2011. **14**(10): p. 1245-6.

7. Dolgin, E., *FDA clearance paves way for computerized ADHD monitoring.* Nat Med, 2014. **20**(5): p. 454-5.

8. Sanefuji, M., et al., *Double-dissociation between the mechanism leading to impulsivity and inattention in Attention Deficit Hyperactivity Disorder: A resting-state functional connectivity study.* Cortex, 2017. **86**: p. 290-302.

9. Jain, A.K., *Data clustering: 50 years beyond K-means.* Pattern Recognition Letters, 2010. **31**(8): p. 651-666.
